# Correlative vs. Causative Relationship between Neonatal Cranial Head Shape Anomalies and Early Developmental Delays

**DOI:** 10.3389/fnins.2017.00708

**Published:** 2017-12-19

**Authors:** Brian T. Andrews, Stefani C. Fontana

**Affiliations:** Department of Plastic Surgery, University of Kansas Medical Center, Kansas City, KS, United States

**Keywords:** early developmental delay, craniosynostosis, deformational plagiocephaly

## Abstract

Deformational plagiocephaly and craniosynostosis are two of the most common neonatal cranial head shape anomalies. Traditionally, both entities were thought to cause aesthetic concerns solely. Recently, many groups have demonstrated that both conditions are strongly associated with developmental delays. The relationship between the abnormal neonatal cranial shape and early developmental delays manifested in both conditions remains poorly understood.

Neonatal cranial head shape anomalies are common and have been attributed to a multitude of factors. Interestingly, despite recent advances in global health care, the incidence of some cranial anomalies has been increasing, making awareness and access to care ever more important. Typically, cranial head shape anomalies are identified in the 1st months of life by primary care providers, who refer these infants to a multi-disciplinary team that specializes in craniofacial disorders. While craniofacial clinics have always been well-suited to manage the aesthetics concerns of cranial head shape anomalies, only recently have concomitant neurodevelopmental delays begun to be addressed in these specialty clinics.

Deformational plagiocephaly (DP), also termed positional plagiocephaly, and craniosynostosis (CS) are two of the most common cranial anomalies encountered in craniofacial clinics. Both conditions can significantly distort the closed cranial vault anatomy in which the developing brain presides. Coincidentally, both conditions are associated with neonatal developmental delays yet it remains indeterminate whether the relationship is causative or correlative. This question has focused recent research efforts to study the impact that the cranial vault shape has on normal brain function and development.

DP is the most common neonatal head shape anomaly affecting 13–48% infants less than 1 year of age (Peitsch et al., 2002). This condition is thought to be exacerbated by prolonged supine positioning. As a result, the skull develops an oblique, parallelogram shape that varies in the severity of the calvarial vault asymmetry (Figure 1). The recent increase in incidence is attributed to the “Back to Sleep Program” introduced by the American Academy of Pediatrics, aimed at combatting the rising numbers of Sudden Infant Death Syndrome (SIDS) (Branch et al., 2015). Interestingly, despite the program's near-universal acceptance by primary care providers, only a minority of infants develop DP, suggesting that this condition is multi-factorial (Habal et al., 2003, 2004). Fortunately, this condition is non-surgical and is managed effectively over several weeks to months with either cranial repositioning interventions or helmet orthosis, though the latter lacks supportive data (Weissler et al., 2016).

**Figure 1:**
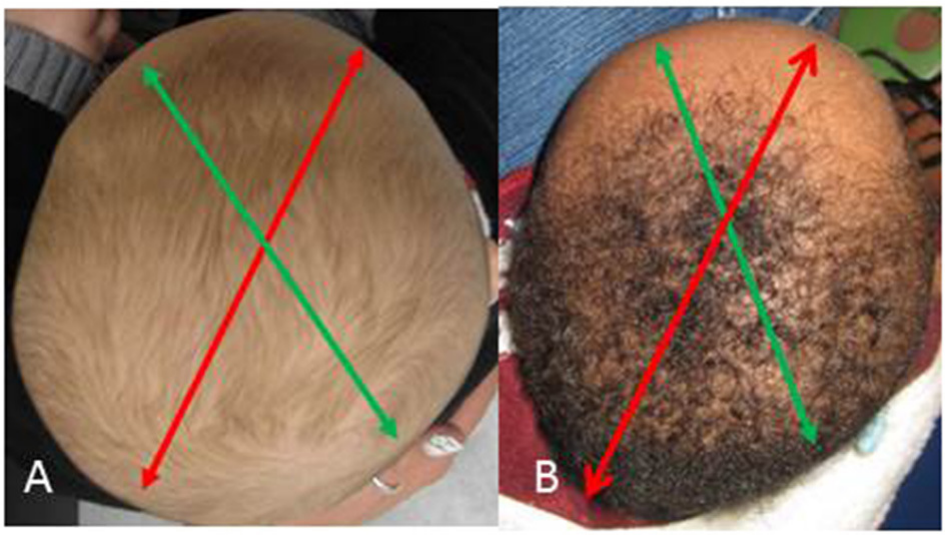
Deformational plagiocephaly. **(A)** Mild right-sided occipital flattening with minimal frontal changes **(B)** Severe right-sided occipital flattening with significant contra-lateral frontal deformity. *Red arrow* indicates long axis and *green arrow* indicates short axis of cranial vault asymmetry.

Many studies have demonstrated that both motor and cognitive delays are associated with DP (Miller and Clarren, 2000; Panchal et al., 2001; Kordestani et al., 2006; Speltz et al., 2010; Collett et al., 2011, 2013; Fontana et al., 2016). The timing of when developmental delays begin remains unknown and most likely delays are manifested as a result of events early in the 1st year of life. More concerning is the recent finds that demonstrate infants with DP are unlikely to developmentally “catch up” to their normocephalic peers when compared at adolescences (Miller and Clarren, 2000). This observation suggests that normal brain development might be both spatially and temporally affected by cranial vault shape during infancy. Although the current medical literature contains many studies showing correlation between DP and developmental delays, no study proves causation. This is an important knowledge gap to consider as DP is a mult-factoral condition (Habal et al., 2003, 2004). Further work is needed to expand on our current knowledge and how best to manage infants with DP.

CS is a much less common disorder (incidence of 1 in 1,800 to 3,000) and results from early fusion of fibrous cranial sutures which serve as growth centers separating immature, growing cranial bones. Premature ossification and union of individual cranial bones, according to Virchow's rule, results in abnormal cranial growth parallel to the fused suture(s). CS requires surgical correction and often cranial vault expansion/remodeling to restore the “normal” infant head shape and aesthetics. Both the timing and the specific operative technique to restore normal cranial anatomy are surgeon-dependent, creating a confounding factor that permeates the literature on this topic.

It is well established that syndromic variants of CS are often associated with neurologic delay. In addition, non-syndromic CS has also been associated with developmental delay but much less commonly. In 2000, Renier et al. first demonstrated that neurodevelopment was delayed in non-syndromic infants with single suture CS (Renier et al., 2000). Prior to this publication most considered CS to be solely an aesthetic problem, yet others quickly confirmed similar observations (Becker et al., 2005; Patel et al., 2014). Interestingly, isolated metopic synostosis has been repeatedly associated with cognitive, behavioral, and language delays (Sidoti et al., 1996; Mendonca et al., 2009). The association between metopic CS and developmental delays poses the question of whether bifrontotemporoparietal brain development is spatially constricted, limiting development in these infants (Figure 2). Another equally plausible scenario is that the lateral frontal lobes are hypoplastic in infants with metopic CS. As a result, poor lateral brain growth results in trigonocephaly. Interestingly, delays are less frequently observed in unilateral and bilateral coronal CS, yet this condition constricts a similar anatomic area but in a different conformation.

**Figure 2:**
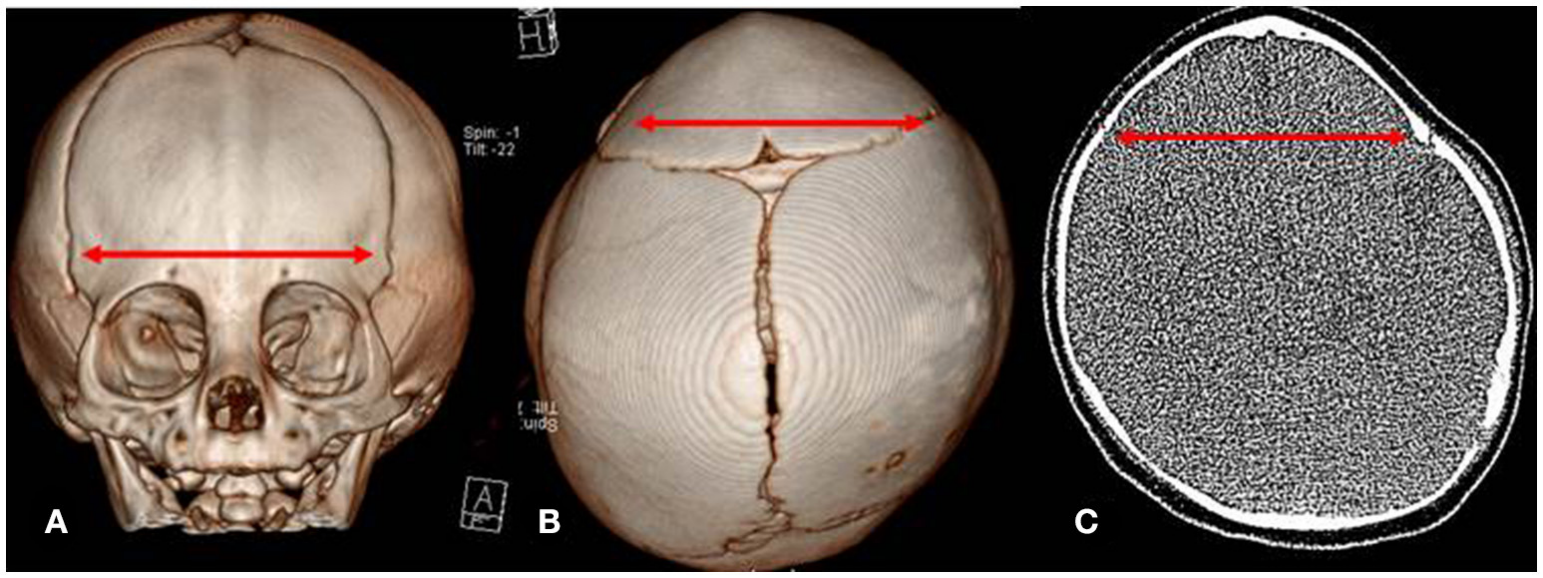
Computed Tomography (CT) images of metopic craniosynostois. **(A)** Anterior view of metopic deformity **(B)** Vertex view of metopic deformity with mild occipital deformational plagiocephaly **(C)** Axial view of metopic deformity. *Red arrow* indicates bifrontotemporoparietal narrowing in each view.

Further complicating the literature is a lack of uniformity in developmental assessment tools. Methods of assessment range from subjective questionnaires completed solely by the parents or guardians (Denver II Developmental Milestones, Ages and Stages questionnaire, and/or MacArthur-Bates Assessment) to objective measures requiring infant participation, such as the Bayley Scales of Infant and Toddler Development III. A common problem with all these measures is that they are limited to certain age ranges and correlation with adolescent and/or adult development is modest at best. One plausible solution is to utilize to utilize a battery of other developmental tests as an initial screening and longitudinal assessment tool (Hashim et al., 2014). Such assessments include the Wechsler Abbreviated Scale of Intelligence, Wechler Fundamentals, Beery-Buktencia Developmental Test of Visual Motor Integration, Behavioral Rating Inventory of Executive Function, and the Behavioral Assessment System for Children. Unfortunately, providers skilled in their implementation are scarce, thus limiting their use and acceptance. As such, no tool has been universally accepted to screen and assess developmental outcomes in craniofacial clinics (Andrews and Fontana, 2016; Fontana et al., 2016).

For several years our craniofacial team has been investigating neurodevelopmental problems associated with both DP and CS. We have conducted prospective studies involving both conditions. Our team has demonstrated that surprisingly, the severity of DP cannot be used to predict either the presence or degree of infant developmental delays (Fontana et al., 2016). Therefore, we recommend that all infants with DP be assessed for delays and, when appropriate, additional supportive services be provided. Furthermore, our studies involving infants with delayed presentation CS (after 1 year of age) demonstrated developmental problems in all infants with cognitive being the most common delay (Fontana et al., 2018). This same study showed that these delays are reversible or improve with cranial vault expansion surgery indicating that the developing brain is capable of repair when abnormal cranial anatomy is corrected. Recently, we have performed serial developmental testing on infants with moderate metopic CS. The majority of these infants demonstrated either a cognitive, language or motor delay at presentation that persisted with observation-only. An age-matched cohort who chose to undergo late cranial vault expansion (greater than 12 months of age) demonstrated improvement in their respective area of developmental delay within 1 year of their surgery (submitted for publication).

There are several problems and criticisms of the medical literature concerning the relationship of developmental delay with DP and CS. First, most studies are retrospective and/or observational in design. This limits the conclusions that can be drawn from their results and at best the relationship remains correlative not causative. Second, as stated earlier, there is no uniformly accepted developmental screening tool to utilize and assess outcomes among differing institutions. Since centers use different developmental assessments it is difficult to compare results amongst institutions. Finally, all current DP and CS studies are from single institutions only with no multi-center studies or meta-analyses available to interpret. To obtain more useful information a large multi-center study or database is required. These knowledge gaps create a large hole in the medical literature. Further work is needed to better understand the relationship between cranial bone growth patterns and infant neurodevelopment. Many groups, including our own, have demonstrated that abnormal skull growth associated with both DP and CS significantly increases the likelihood of developmental delays. While the cause of these delays occur is yet poorly understood, there does seem to be a trend toward normalization in infants with CS who have surgical correction.

## Author contributions

Both BA and SF were involved in the data collection, review of literature and the writing of this paper.

### Conflict of interest statement

The authors declare that the research was conducted in the absence of any commercial or financial relationships that could be construed as a potential conflict of interest.
